# Application of intelligent packaging for meat products: A systematic review

**DOI:** 10.1002/vms3.1017

**Published:** 2022-12-26

**Authors:** Seyedeh Mahsa Khodaei, Majid Gholami‐Ahangaran, Iraj Karimi Sani, Zahra Esfandiari, Hadi Eghbaljoo

**Affiliations:** ^1^ Department of Food Science and Technology Nutrition and Food Security Research Center School of Nutrition and Food Science Isfahan University of Medical Sciences Isfahan Iran; ^2^ Department of Poultry Diseases Faculty of Veterinary Medicine Shahrekord Branch, Islamic Azad University Shahrekord Iran; ^3^ Department of Food Science and Technology Faculty of Agriculture Urmia University Urmia Iran; ^4^ Division of Food Safety and Hygiene Department of Environmental Health Engineering School of Public Health Tehran University of Medical Sciences Tehran Iran

**Keywords:** integrity and freshness indicators, intelligent packaging, meat, radio frequency identification tags, shelf life extension, time temperature indicators

## Abstract

**Background:**

Today, in response to consumer demand and market trends, the development of new packaging with better performance such as intelligent packaging has become more important. This packaging system is able to perform intelligent functions to increase shelf life, increase safety and improve product quality.

**Objectives:**

Recently, various types of packaging systems are available for meat products, especially cooked, fresh and processed meats. But because meat products are very perishable, monitoring their quality and safety in the supply chain is very important. This systematic article briefly reviews some of the recent data about the application of intelligent packaging in meat products.

**Methods:**

The search was conducted in Google Scholar, Science Direct, Elsevier, Springer, Scopus, and PubMed, from April 1996 to April 2021 using a different combination of the following keyword: intelligent packaging, and meat.

**Results:**

The results showed that the intelligent packaging presents several benefits compared to traditional packaging (e.g., antimicrobial, antioxidant, and shelf life extension) at the industrial processing level. Thus, these systems have been applied to improve the shelf life and textural properties of meat and meat products.

**Conclusions:**

It is necessary to control the number of intelligent compounds that are included in the packaging as they clearly influence the quality and nutritional properties as well as the final cost of the food products.

## Graphical abstract

Today, in response to consumer demand and market trends, the development of new packaging with better performance such as intelligent packaging has become more important. In this review showed that the intelligent packaging presents several benefits compared to traditional packaging (e.g., antimicrobial, antioxidant and shelf life extension) at the industrial processing level.

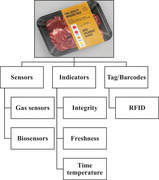

## 1 INTRODUCTION

The spoilage of food and food products, for example, meat, is a main concern in public health (Rahimi et al., 2012). Most of common zoonotic pathogens transfer via food chain to human and induce health risk (Gholami‐Ahangaran et al., 2015). The utilisation of compounds or technology that can inhibit or delay the spoilage is very important in food sciences (Gholami‐Ahangaran et al., 2022). The use of biological compounds to preserve the nature of food is not always successful (Gholami‐Ahangaran et al., 2019). In general, packaging protects food from environmental factors such as moisture, light, oxygen, microorganisms, dust and mechanical stress. Due to the growing demand of consumers for foods that are minimally processed and ready to eat, as well as due to the globalisation of the food industry, it is necessary to maintain the freshness and optimal quality of food at long times, and this leads to increasing growth tendency to novel packaging (Kerry et al. 2006).

Traditional packaging of fresh meat is done to prevent contamination, delay spoilage of the product, and allow the activity of some enzymes to improve the tenderness of meat texture, reduce weight loss, and ensure the formation of oxymyoglobin pigment (instead of metmyoglobin) for the bright red colour (Panea et al., 2014). However, today, with the advancement of technology and increasing demand from consumers and industry, traditional packaging methods are not able to provide meat products that have longer shelf life, and are safer and healthier, easier to consume, in line with the environment, and reduce food waste (Ahmed et al., 2018). In response to these challenges, a new generation of packaging called intelligent packaging has been introduced to the market (Choi et al., 2014).

Intelligent packaging is one of the new packaging technologies in recent years for various foods, including meat and meat products. Intelligent packaging informs the consumer about the status of the food by understanding some of the characteristics of the food in the package or the characteristics of the environment (Panea et al., 2014). The most important intelligent packaging tools are sensors and indicators. Intelligent packaging systems can detect and warn of product quality changes during storage. Sensors and detectors and radio frequency detection systems (RFID) are the tools used in intelligent packaging (Kerry et al. 2006).

However, there is a lack of an overview that summarises the characteristics of meat products packed in intelligent packaging. Therefore, the purpose of this study is to review the application of intelligent packaging in the meat industry such as red meat, poultry, chicken, fish and processed meat from 1996 to 2021. Moreover, current challenges in intelligent packaging were identified that can boost their technological characteristics.

### 1.1 Sensors

#### 1.1.1 Gas sensors

Gas sensors determine the gases of the space of packages and can quickly and cheaply determine the quality of the meat product (Kerry et al., 2006). Therefore, intelligent packages equipped with gas sensors have been designed. Visual chemical sensors are among these gas sensors that are able to detect the onset of spoilage by sensing gases resulting from microbial spoilage such as hydrogen sulphide (H_2_S) or carbon dioxide (CO_2_) (in red meats) or volatile amines (in fishes) in the packaged space of meat products (Pereira et al., 2021). These gases are important to be monitored during packaging due to, for example, H_2_S, and volatile amines are produced during meat spoilage by microorganisms (Casaburi et al., 2015). The response of gas sensors correlates with bacterial growth patterns in meat samples, thus enabling ‘real‐time’ monitoring of spoilage in different types of meat (Pacquit et al., 2006).

In visual chemical sensors, for example, pH‐sensitive sensors based on the fluorescence system can be used in conjunction with the sensors. Oxygen sensors based on fluorescence are another types of gas sensors, which have been used to measure gases in the headspace of meat products (Ahmed et al., 2018).

#### 1.1.2 Biosensors

Rapid, accurate and online understanding is a requirement for on‐site analysis of contaminants, determination and detection of pathogens and control of food quality parameters after processing. In general, a biosensor is a compact analyser that detects, records, and transmits information about biochemical reactions (Badihi‐Mossberg et al., 2007). This intelligent device has two primary components: a bioreceptor that detects target analytes and a transducer that converts biochemical signals into measurable electrical responses (Yam et al. 2005). A bioreceptor is an organic or biological substance, such as an enzyme, antigen, microbe, hormone or nucleic acid (Biji et al., 2015). The transducer, based on the measured parameters, can exist in different forms such as electrochemical, optical, acoustic (Senturk et al., 2018).

### 1.2 Indicators

#### 1.2.1 Integrity indicators

Integrity indicator is a type of detector that is used to determine the breakdown of packages, and show the qualitative information related to packaging in the form of colour changes. The damage and of leakage in the packages is one of the most common damages to packages containing meat products, which can be detected by the above‐mentioned indicators. Most of the indicators that detect leakage in the package are in fact detectors that show the presence of oxygen in the package through a leak. In these packages usually, the increase in the amount of oxygen can indicate damage and leakage in the package. In fact, oxygen enters the package through the orifice, so visual oxygen detectors are used (Ahmed et al., 2018).

#### 1.2.2 Freshness indicators

Freshness indicators provide direct quality information about the product as a result of microbial growth or chemical changes in the food product. Microbiological quality may be detected by reactions between encapsulated markers and microbial growth metabolites. Changes in the concentrations of organic acids such as n‐butyrate, L‐lactic acid, D‐lactate and acetic acid during storage as potential metabolites for a number of meat products provide information about the freshness of product. These microbial metabolites are produced during growth, activity and metabolism of microorganisms. They have an effect on the freshness indicators of meat products (Casaburi et al., 2015). Colour‐based pH sensing is used as indicators of these microbial metabolites (Rokka et al., 2004).

#### 1.2.3 Time temperature indicators (TTI)

Temperature is usually the most important environmental factor that like microbial growth, affects the kinetics of physical and chemical degradation in food products. Time‐temperature indicators (TTIs) are very useful in the food industry because they can alert the consumer when food is exposed to inappropriate temperatures. TTIs are usually small self‐adhesive labels that are affixed to shipping containers or single packages. These labels have visual indications of the temperature background during distribution and storage, which are especially useful for warning of unsuitable temperatures for refrigerated or frozen food products. These detectors are also used to estimate the remaining shelf life of perishable products. All of the commercially available TTIs have the potential to be used in meat products (Vaikousi et al., 2009).

### 1.3 Tag/barcodes

A barcode is a machine‐readable storage database that operates on the optical phenomenon of bars of regular width and thickness. If pathogenic bacteria grow inside the package during the storage, it can be detected by the bar code and as a result, the colour changes and the bar code becomes unreadable (Kerry et al., 2006).

#### 1.3.1 Radio frequency detection (RFID)

Radio frequency detection systems are one of the most diverse technologies for automatic detection or identification. RFID systems have many advantages in the production, warehousing, distribution and retail chains of meat products. The reduced maintenance costs, safety and improvement of the quality of the product and prevention of the return of the product are some benefits of RFID systems (Kerry et al., 2006).

### 1.4 Need for intelligent packaging of meat products

Meat is one of the most perishable food groups, and the correct packaging, in addition to increasing the shelf life, plays an important role in reducing waste and increasing the level of public health by reducing pollution caused by the use of unsanitary and inappropriate products. Meat spoilage is mainly caused by microbial degradation and lipid oxidation due to its high water activity (a_w_) and fat content. Spoilage of meat products can lead to quality loss such as colour change, off‐flavour, loss of crispness, and change in pH, which ultimately causes in consumer rejection and economic losses (Ahmed et al., 2018; Wojnowski et al., 2017).

Off‐odour is one of the key indicators of meat spoilage. These odours are generally attributed to the accumulation of volatile compounds in the packaging headspace, particularly sulphur‐containing compounds, biogenic amines and other low‐molecular‐weight VOCs, which are mainly caused by microbial activity on proteins, amino acids, and carbohydrate substrates (Luo et al., 2022). *Br. thermosphacta* is one of the important bacterial species responsible for off‐odour and *S. putrefaciens* is known to produce hydrogen sulphide during meat spoilage (Casaburi et al., 2015).

The primary method for detecting meat spoilage is microbiological testing through the total count of bacteria and/or microbial species causing spoilage, including *Acinetobacter* spp., *Brochothrix thermosphacta*, Enterobacteriaceae, *Lactobacillus* spp., *Pseudomonas* spp. and *Shewanella* (Wojnowski et al., 2017). Sensory analyses based on colour change, off‐ odour and sliminess are common (Ahmed et al., 2018). These methods are time‐consuming, laborious and require special expertise. Therefore, the development of new rapid techniques that can reflect meat quality in real‐time and detect its spoilage is valuable for the meat industry. Gas chromatography is the most common method to determine the volatile compounds from meat spoilage. This technique is relatively expensive and requires instrumental expertise (Luo et al., 2022).

**FIGURE 1:**
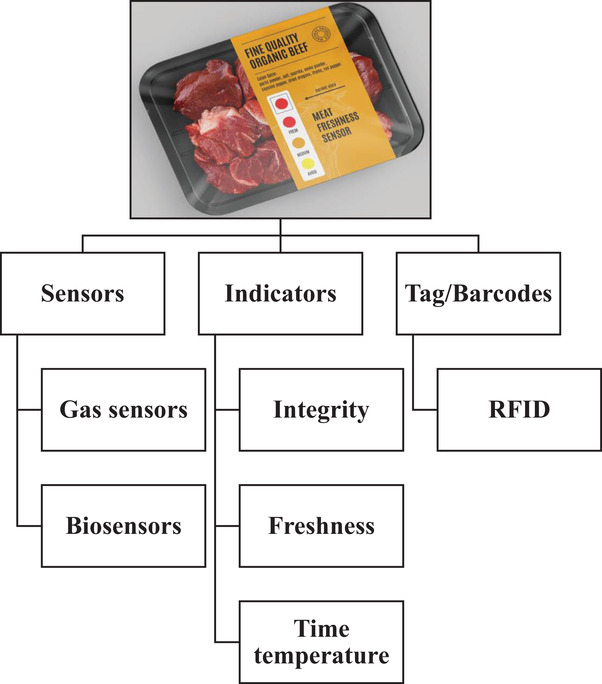
Intelligent packaging used for meat products

The packaging of meat products is to prevent contamination, delay spoilage and allow some enzyme activities to improve softness, dehydration, fat oxidation and colour change. Conventional and traditional food packaging with the aforementioned basic functions is no longer sufficient in the food chain due to increasing concerns in product safety, food waste production, changing consumer lifestyles and emerging marketing trends. Innovative techniques with advanced functionalities are required. During the last two decades, intelligent packaging systems have been developed. Intelligent packaging (Figure 1) facilitates the flow of information during transport or at the home. This information can be converted into visual information through barcodes or indicators. For example, an intelligent packaging system can show the decline of freshness over time, temperature fluctuations during storage in different environmental conditions, and changes in gas composition in the packaging space, or the distribution date of the product (Luo et al., 2022).

## 2 MATERIALS AND METHODS

### 2.1 Search strategy

In this systematic review, the specialised databases, namely, Google Scholar, Science Direct, Elsevier, Springer, Scopus and PubMed, were used for the literature search from April 1996 to April 2021, with the purpose of limiting the search to the latest findings, using different combinations of the following keywords: intelligent packaging and meat. In Google Scholar, Direct, Elsevier and Springer, we used the following search equation strategy: (intelligent AND meat products). The search equation used in Scopus and PubMed was: ‘intelligent’ AND, meat.

### 2.2 Selection criteria

Articles were organised by the application of intelligent packaging in meat products. Three members of the team (H. Eghbaljoo, S.M. Khodaei and M. Gholami Ahangaran) extracted information about the characteristics of the articles. The information extracted from the articles included intelligent packaging applications in meat products such as red meat, poultry, chicken, fish and processed meat. After that, the quality evaluation and selection were performed by three authors (H. Eghbaljoo, I. Karimi Sani and Z. Esfandiari) who independently worked according to the main criteria of PICO (Population, Intervention, Comparison, and Outcome) (Table 1).

**TABLE 1:** PICO (Population, Intervention, Comparison, Outcome) criteria for inclusion of studies

Parameter	Inclusion criteria
Population	Studies accomplish meat, poultry and fish
Intervention	Treatment with intelligent packaging
Comparison	Intelligent packaging vs. control
Outcome	Intelligent and active packaging in meat products

### 2.3 Data handling, analyses and extraction

The inclusion criteria for handling of studies were outlined according to PRISMA guidelines and used were the following: (1) intelligent packaging in meat products; (2) nanoparticles (NPs) in meat products and (3) studies with significant results collected via statistical analysis. The exclusion criteria used were as follows: (1) studies written in the English language; (2) the use of intelligent packaging, instead of intelligent; (3) studies without controls; and (4) the assessment of the efficiency of modern packaging in meat products. After removing duplicates, the title and abstract of each article were reviewed by one member of the team (H.E). After that, acceptability for inclusion was analysed based on the following: (1) reading the title and abstract by three authors (H. Eghbaljoo Gharehgheshlaghi, S.M. Khodaei and M. Gholami Ahangaran); and (2) reading the full text by three authors (H. Eghbaljoo Gharehgheshlaghi, I. Karimi Sani and Z. Esfandiari) (Figure 1). Data were extracted by one author (H.E.) into forms on Microsoft Excel 2016. Article selection and data extraction differences were resolved through discussion. The main results of the selected articles were arranged according to the applications of intelligent packaging in the meat industry.

## 3 RESULTS

### 3.1 Study identification and selection

**FIGURE 2:**
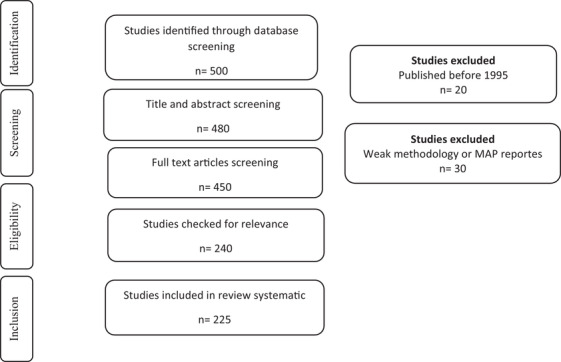
PRISMA flow chart for studies related with intelligent packaging in meat products

Of the 300 full texts reviewed, 138 relevant articles were identified, which was in agreement with our inclusion and exclusion criteria. The selected articles were grouped into intelligent packaging and nanoparticles in meat products. The complete process is shown in Figure 2, which is based on a PRISMA flow chart.

### 3.2 Intelligent packaging

Types of intelligent packaging and commercial applications for meat products are summarised in Table 2. The results of applications of intelligent packaging in meat products of selected articles and their main results are presented in Table 3. In total, 84 articles were identified and characterised the effects of intelligent packaging for meat products. According to the results, intelligent packaging described the microbial quality and is an effective spoilage indicator by evaluating their reaction to the metabolites produced during the growth of microorganisms or during chemical changes within the meat products.

**TABLE 2:** Types of intelligent packaging and commercial applications for meat products

Indicator	Commercial name	Company	System
Integrity indicators	Timestrip®	Timestrip Ltd.	Time indicator label
	Novas®	Insignia Technologies Ltd.	Time indicator label
	Best‐by®	FreshPoint Lab.	Time indicator label
	Ageless Eye®	Mitsubishi Gas Chemical Inc.	Gas indicator tablet
	Tell‐Tab I	MPAK	Gas indicator tablet
	O_2_Sense	Freshpoint Lab.	Gas indicator tablet
Freshness indicators	Fresh Tag®	COX Technologies	Colourimetric indicator
	SensorQ®	DSM NV and Food Quality Sensor International Inc.	pH‐sensing indicator
	Raflatac	VTT and UPM Raflatac	Colourimetric indicator (silver nanolayers)
	Food Sentinel	System SIRA Technologies Inc.	Biosensor (barcode)
	Toxin Guard®	Toxin Alert Inc.	Biosensor (film)
	RipeSense	RipSense^TM^ and ort Research	‐
Time temperature indicators (TTI)	3 M Monitor Mark®	3 M Company	Fatty acid ester TTI
	Keep‐it®	Keep‐it Technologies	Chemical TTI
	Fresh‐Check®	Temptime Corp.	Polymerisation reaction TTI
	VITSAB®	VITSAB International AB	Enzymatic TTI
	OnVu®	Freshpoint and Ciba	Photochemical reaction TTI
	TopCryo®	TRACEO	Microbiological TTI
	FreshCode®	Varcode Ltd.	Barcode based label TTI
	Tempix®	Tempix AB	Barcode based label TTI
	Cook‐Chex	Pymah Corp.	‐
	Timestrip ®	Timestrip Plc	‐
	Colour‐Therm	Colour‐Therm	‐
	MonitorMark^TM^	3 M ^TM^ Minnesota	‐
	Onvu^TM^	Ciba Specialty Chemical and	‐
	Fresh‐Check®	Temptime Corp.	‐
	Thermax	Thermographic Measurements Ltd.	‐
	Novas®	Insignia Technologies Ltd	‐
	Best‐by®	FreshPoint Lab	‐
	CheckPoint®	Vitsab	‐
Radio frequency identification tags (RFID)	Easy2log®	CAEN RFID Srl	TT sensor tag
	CS8304	Convergence Systems Ltd.	TT sensor tag
	TempTRIP	TempTRIP LLC	TT sensor tag
	Intelligent box	Mondi Plc	Box with integrated TT sensor tag
	Intelligent fish box	Craemer Group GmbH	Box with integrated TT sensor tag
	AMS SL13A	‐	Temperature, expandable with external sensor
	CAEN RFID easy2log RT0005ET	‐	Temperature
	Intelleflex TMT‐8500	‐	Temperature
	SecureRF Lime Tag 2.0 Sensor	‐	Temperature, expandable to pH‐level, relative humidity and shock sensing

**TABLE 3:** Summary of intelligent packaging applications for meat products

Indicator	Food product	Function	References
Freshness indicators	Poultry meat	Carbon dioxide colourimetric indicators	Saliu and Pergola (2018)
	Meat	Antimicrobial	Liu et al. (2016)
	Minced beef	Alizarin colourimetric indicator	Ezati et al. (2019)
	Lean pork	pH dye‐based indicator Freshness via colour change	Chen et al. (2019)
	Tilapia	Indicator based on polyaniline	Wang et al. (2018)
	Fish	Changes in pH and thiobarbituric acid content	Morsy et al. (2016)
	Skinless chicken breast	A colourimetric mixed‐pH dye‐based indicator Carbon dioxide (CO_2_) was used as a spoilage metabolite because the degree of spoilage was related to the amount of increased CO_2_	Rukchon et al. (2014)
	Meat and seafood	Increases in TVB‐N and increases in bacterial colonies (TACC), key indicators of spoilage	Dudnyk et al. (2018)
	Fish products	pH‐sensitive dye bromocresol green	Chun et al. (2014)
	Fish	A novel colourimetric film based on polysaccharide	Huang et al. (2019)
	Chicken‐breast	Tyvek® sheet and RGB colour analysis	Lee et al. (2019)
	Meat and seafood: catfish fillets (*Ictalurus punctatus*)	Paper‐based and pH‐sensitive detector	Etebari Alamdari et al. (2020)
	Beef	pH indicators	Kuswandi et al. (2017)
	Shrimp and crab	Amine‐responsive cellulose‐based ratiometric fluorescent materials	Jia et al. (2019)
	Red and white meat	Biogenic amines	Vinci and Antonelli (2002)
	Fresh beef, pork, and chicken meat	Biogenic amines and volatile basic nitrogen	Min et al. (2007)
	Fish	A novel colourimetric indicator film based on gelatin/polyvinyl alcohol incorporating mulberry anthocyanin extracts	Zeng et al. (2019)
	Packed fish: cod	Ammonium detection method	Heising et al. (2012)
	Rainbow trout	PH‐sensitive Indicator	Rastiani et al. (2019)
	Fish	Polyaniline film	Kuswandi et al. (2012)
	Fish	Tetraphenylethylene‐functionalised polyaniline sensing label	Liu et al. (2020a,b)
	Fish	Sol‐gel matrix	Liu et al. (2020a,b)
	Fish	pH sensitive dye visible colour changes to the spoilage volatile compounds total volatile basic nitrogen (TVB‐N)	Pacquit et al. (2007)
	Fish	Volatile amine	Pacquit et al. (2006)
	Pork sausages	Optoelectronic nose	Salinas et al. (2014)
	Spanish mackerel (fish)	pH indicator	Sun et al. (2015)
	Sea bream	Optoelectronic nose	Zaragoza et al. (2013)
	Atlantic salmon	Optoelectronic nose	Zaragoza et al. (2014)
	Ground meat and salmon	Optical and electrochemical dye sensors based on 4‐(dioctylamino)‐4′‐(trifluoroacetyl)azobenzene	Lin et al. (2015)
	Fish	*Brassica oleraceae* (red cabbage) as a visual indicator	Silva‐Pereira et al. (2015)
	Chub	Colourimetric sensor array	Huang et al. (2011)
	Chicken	Colourimetric sensor array with AdaBoost‐OLDA classification algorithm	Chen et al. (2014)
	Chicken	Tricyanofuran hydrazone dyes	Khulal et al. (2017)
	Chicken	Fabricated colourimetric sensor array	Khulal et al. (2016)
	Chicken	Colourimetric sensor array	Salinas et al. (2014)
	Boiled marinated turkey meat	Chromogenic sensor array	Salinas et al. (2014)
	Chicken	Colourimetric sensor array	Chen et al. (2016)
	Cooked chicken	Colourimetric sensor array	Kim et al. (2016)
	Pork	Portable electronic nose (E‐nose) based on a colourimetric sensor array	Li et al. (2014)
	Pork	Integrating hyperspectral imaging and colourimetric sensor	Li et al. (2015)
	Chicken, pork, beef, fish and shrimp	Portable optoelectronic nose	Li and Suslick (2016)
	Pork	pH indicator	Choi et al. (2017)
	Tuna and beef	Solid polymer substrates and coated fibres containing 2,4,6‐ trinitrobenzene motifs	Pablos et al. (2015)
	Yao‐meat pork	Nanoporous colourimetric sensor arrays	Xiaowei et al. (2016)
	Yao‐meat pork	Colourimetric sensor array based on nine natural pigments	Xiaowei et al. (2015)
	Yao‐meat pork	Colourimetric sensor	Huang et al. (2014b)
	Pork	Colourimetric gas sensor array based on natural pigments	Huang et al. (2014a)
	Chicken breast fillets	Colourimetric sensor array	Urmila et al. (2015)
	Red meat	Optical detection of amines	Schaude et al. (2017)
	Beef steaks	FreshCase technology	Yang et al. (2016)
	Fish and meat	Fluorescent nanofibres	Che et al. (2008)
Time temperature indicators (TTI)	Ground beef	Antimicrobial	Kim et al. (2013)
	Fish		Giannakourou et al. (2005)
	Ground beef	Antimicrobial	Kim et al. (2012)
	Pork	Antimicrobial	Morelli et al. (2012)
	Sliced ham	Antimicrobial	Derens‐Bertheau et al. (2015)
	Beef products	Antimicrobial	Choi et al. (2014)
	Bogue fish	Amylase type	Yan et al. (2008)
	Ground beef and spiced cooked chicken slices	Antimicrobial and safety	Ellouze and Augustin (2010)
	Yellowfin tuna slices	Safety monitoring	Tsironi et al. 2008
	Chilled fish	Shelf life control	Taoukis et al. (1999)
	Minced meat	Antimicrobial	Vaikousi et al. (2009)
	Meat and poultry products	Antimicrobial	Labuza and Fu (1995)
	Grounded pork patty	Antimicrobial	Chun et al. (2014)
	Buffalo meat	Colourimetric indicator sensor based on bromophenol blue sensitive to total volatile basic nitrogen (TVBN)	Shukla et al. (2015)
	Meat and meat products	Antimicrobial	Kreyenschmidt et al. (2010)
	Beef sirloin	Antimicrobial	Han et al. (2012)
	Broiler chicken cuts	Improvement of microbiological shelf‐life	Smolander et al. (2004)
	Broiler chicken cut	Sticker sensor based on methyl red	Kuswandi et al. (2014)
	Meat products	Enzymatic validation of pasteurisation	Brizio and Prentice (2015)
	Chilled boneless chicken breast	Quality control	Brizio and Prentice (2014)
	Atmosphere packed gilthead seabream fillets	UV activatable photochemical TTI	Tsironi et al. (2011)
	Chilled vacuum‐packed grouper fillets	Antimicrobial	Hsiao and Chang (2016)
	Fresh salmon (*Salmo salar*)	Antimicrobial	Simpson et al. (2012)
	Turbot sashimi	Tyrosinase‐based TTI	Xu et al. (2017)
	Meat	Discolouration process under dynamic temperature conditions	Albrecht et al. (2020)
	Chicken breast meat	Antimicrobial	Park et al. (2013)
Radio frequency identification tags (RFID)	Meat	Freshness monitoring	Eom et al. (2014)
	Pork	Freshness monitoring	Sen et al. (2013)
	Meat	Freshness monitoring	Townsend and Mennecke (2008)
	Chilled meat	Freshness monitoring	Swedberg (2011)

## 4 DISCUSSION

In industrialised countries, food companies make large investments in the use of novel packaging technologies, and it is believed that if the packaging is suitable in different ways and can provide satisfy the consumers, will lead to more product sales and faster return on investment with appropriate profits (Ahmed et al., 2018).

Intelligent packaging systems are systems that can detect, signal and warn of food product quality changes during storage. Sensors and indicators [e.g., integrity detectors, time‐temperature (TTI) detectors and radio frequency detection (RFID) systems] can be used in intelligent packaging (Kerry et al., 2006). In order to develop the commercial application of these technologies, the knowledge and awareness of industry about their benefits, increase the efficiency of these technologies, paying attention to the economic aspects of their use and increase consumer acceptance. In this article, the results of some research related to these new technologies and their applications for the packaging of meat and meat products were presented (Panea et al., 2014).

The maintaining of integrity, retarding the spoilage of meat products, prolonging the shelf life of meat, improving the quality properties of meat and meat products, retard the lipid oxidation, etc. were some of the functional properties of intelligent packaging.

Ellouze and Augustin (2010) and Park et al. (2013) employed a biological TTI from lactic acid bacteria strains in the chicken slices and ground beef and chicken breast packaged under modified atmosphere and observed this TTI helped to monitor the spoilage in the samples and therefore, employed as a quality and safety indicator of the meat products.

Rukchon et al. (2014) employed a colourimetric pH indicator for real‐time monitoring of freshness of skinless chicken breast. This indicator was a mixture of bromothymol blue, methyl red and a mixture of bromothymol blue, bromocresol green and phenol red.

Pundir et al. (2010) developed a biosensor based on xanthine that is used to monitor the freshness of meat products. Hernández‐Cázares et al. (2010) developed an enzyme sensor by combining O_2_ electrodes and xanthine oxidase to indicate the freshness of pork by measurement of hypoxanthine content. Smiddy et al. (2002) estimated lipid oxidation in cooked chicken and raw and cooked beef employing O_2_ sensors and showed that these sensors are suitable for the measurement of O_2_ in meat packages and predicting the quality of processed meats.

In recent years, several studies have been focused on the development of rapid methods to monitor microbial breakdown and real‐time freshness of fish and seafood products, using intelligent packaging. During spoilage, fish releases a variety of basic volatile metabolites, which are detected with sensors. Silva‐Pereira et al. (2015) reported a system for pH monitoring based on corn starch, chitosan and red cabbage extract during fish spoilage. At the first steps of degradation, the colour changed from colourless to blue and finally to yellow when the samples were completely spoiled.

Similarly, several studies have been focused on the colourimetric indicators used in chicken. For example, Chen et al. (2014) developed pH indicators for the analysis of chicken meat. In addition, Salinas et al. (2014) in the various researches explored the monitoring of chicken and boiled marinated turkey meats using intelligent indicators.

Some studies for monitoring pork and buffalo meat freshness were published (Choi et al., 2017; Li et al., 2014; 2015; Shukla et al., 2015) with a colourimetric sensor sensitive to TVB‐N released during meat storage.

A much simpler sensor was developed by Pablos et al. (2015) to detect beef freshness. These sensors were based on colour changes, when in contact with the atmosphere inside the package.

Similar findings have been reported in the literature in regards to monitoring raw and processed meat (Kerry et al., 2006) as a tool to reduce their wastes and prolonging the shelf life of products.

So far, various nanoparticles have been used in the intelligent packaging of meat and meat products. Many studies have been done on the antimicrobial effect of gold and silver nanoparticles on various microorganisms. Silver nanoparticles also reduced the microbial load of beef packaged under the modified atmosphere. Morsy et al. (2014) reported edible films made from pullulan incorporated with essential oils and AgNPs can maintain the quality of processed meat and poultry products. Similarly, commercial films coated with AgNPs could be to increase the shelf life of Turkey meat (Deus et al., 2017).

Continuous research seems to be needed to access the benefits and capabilities of intelligent packaging for meat and meat products. The scope of this research may be more appropriate in cases such as modelling of interactions between foods and microorganisms and their metabolites in different storage conditions, the relationship between the detection of spoilage and the sensory quality of food, suitable sensors and detectors, the behaviours and characteristics of tools used for intelligent packaging in different parts of the production, warehousing and distribution chain, as well as a more complete understanding of the sensitivities and reliability of intelligent packaging and their tools (Ahmed et al., 2018, Kerry et al., 2006).

The potential benefits of intelligent packaging for meat and muscle products are varied. Paying attention to the positive effects of this type of packaging on the quality, safety and health of food in different stages, we must also pay attention to its economic and marketing aspects (Panea et al., 2014).

The increasing consumer information and awareness of consumers and their demands are among the factors that force manufacturers and researchers to innovate, develop and optimise modern packaging technologies (Ahmed et al., 2018). Different forms of intelligent packaging such as the use of oxygen sensors, freshness and time‐temperature indicators are the answers that researchers and scientists have developed these demands. If the necessary coordination between efficiency and usefulness is established with the economic aspects of the use of intelligent packaging, in the future, the use of these new technologies for packaging various foods, including meat and meat products, will be inevitable (Kerry et al., 2006).

## 5 CURRENT CHALLENGES IN INTELLIGENT PACKAGING

Food manufacturers should educate about food safety of intelligent packaging. Current regulations require that migration rate below from packaging materials to food. The acceptability of intelligent packaging techniques is considered from the point of view of migration. These techniques can be divided into two groups: Group 1, consists of external indicators fixed on the outer surface of a package, such as time‐temperature indicators and Group 2, consists of internal indicators intended to be placed in the main part of a package, such as oxygen and carbon dioxide indicators. The migration does not occur in group 1 indicators because there is no direct contact of the indicator with the food product. Group 2 indicators are not intended for direct contact with packaged foods. However, they are placed in the free space of a package or fixed on the inner surface (Han et al., 2005; Kalpana et al., 2019).

It seems that extensive research is needed to access the advantages and capabilities of intelligent packaging. The fields of this research can be more appropriate in the modelling of the interactions between foods and microorganisms and their metabolites in different storage conditions, a better understanding of the relationships between spoilage detection and the sensory quality of food, finding suitable sensors and indicators, increasing information about the characteristics of the used tools for intelligent packaging, storage and distribution chain, understanding of the sensitivities and confidence levels related to intelligent packaging. The potential advantages of intelligent packaging are many and varied. Of course, in addition to the positive effects of intelligent packaging on the quality, safety and health of food should also be paid attention to its economic and marketing aspects. The awareness of consumers is force manufacturers and researchers to innovate and develop and optimise modern packaging technologies. Various forms of intelligent packaging, such as oxygen sensors, freshness and spoilage indicators and time‐temperature detectors, are answers that researchers and scientists have devised to meet the aforementioned demands. If the necessary coordination between efficiency and usefulness is established with the economic aspects of the use of intelligent packaging, it will be inevitable to use these technologies in the future.

## 6 CONCLUSIONS

In this article, the results of some research and articles related to new technologies and their applications for the packaging of meat and meat products are presented. Monitoring the quality and spoilage of fresh meat products is essential in order to reduce the incidence of foodborne illness and reduce the production of meat waste throughout the supply chain. However, traditional packaging systems are able to provide few services in the field of supply chain monitoring. But new intelligent packaging systems with the aim of monitoring the quality of packaged meat or its environment are advancing towards providing innovative solutions in the industry of production and supply of meat products. So, a variety of commercial freshness, temperature‐time, integrity and radio frequency detectors with intelligent concepts, in order to improve storage conditions and reduce waste of fresh and safer meat products, have been introduced to the food market. However, each of these methods has advantages and disadvantages, which affect the performance and efficiency of the system. Therefore, it is necessary to control the number of intelligent compounds that are included in the packaging as they clearly influence the quality and nutritional properties as well as the final cost of the food products.

## AUTHOR CONTRIBUTIONS

H. Eghbaljoo, S.M. Khodaei and Z. Esfandiari contribute in supervision, investigation, validation and methodology. I. Karimi assists in search and investigation. H. Eghbaljoo, Z. Esfandiari and M. Gholami‐Ahangaran contribute in methodology, analysis of data and writing the original draft manuscript and reviews.

## CONFLICT OF INTEREST

The authors declare no conflicts of interest.

## FUNDING

The authors did not receive any supportive finances.

### ETHICS STATEMENT

In this manuscript, all the ethical principles related to writing a review article, including maintaining trustworthiness and avoiding plagiarism, have been observed.

### PEER REVIEW

The peer review history for this article is available at https://publons.com/publon/10.1002/vms3.1017.

## Data Availability

The data are in access of corresponding author who replies after request.
